# Results of hearing screening of school-age children in Bishkek, Kyrgyzstan

**DOI:** 10.1017/S1463423620000183

**Published:** 2020-06-10

**Authors:** Piotr Henryk Skarżyński, Weronika Świerniak, Elżbieta Gos, Irina Pierzyńska, Adam Walkowiak, Katarzyna Beata Cywka, Kinga Wołujewicz, Henryk Skarżyński

**Affiliations:** 1 Department of Teleaudiology and Screening, World Hearing Center, Institute of Physiology and Pathology of Hearing, Warsaw/Kajetany, Poland; 2 Heart Failure and Cardiac Rehabilitation Department, Faculty of Medicine, Medical University of Warsaw, Warsaw, Poland; 3 Institute of Sensory Organs, Warsaw/Kajetany, Poland; 4 Department of Oto-Rhino-Laryngosurgery, World Hearing Center, Institute of Physiology and Pathology of Hearing, Warsaw/Kajetany, Poland; 5 Centre of Hearing and Speech Medicnus, Kajetany, Poland; 6 Department of Implants and Auditory Perception, World Hearing Center, Institute of Physiology and Pathology of Hearing, Warsaw/Kajetany, Poland

**Keywords:** hearing loss, hearing screening, prevalence, pure-tone audiometry, school screening

## Abstract

**Aim:**

To gauge the prevalence of hearing loss in school children in Bishkek, Kyrgyzstan, and refer pupils with positive results for further diagnostic testing.

**Background:**

According to WHO data, hearing disorders are common in school-age children. Screening for hearing loss is an important preventative tool, helping to avoid further complications. Expenditure that supports early child development can reduce future outlay on health care and social services; it can eliminate disability problems, education deficits, and social maladaptation in later adult life.

**Methods:**

Pure-tone air-conduction hearing thresholds were obtained at 0.5–8 kHz. The results of the hearing screening examination were regarded as positive if pure-tone thresholds were higher than 20 dB HL in one or both ears at one or more of the test frequencies. Data were also obtained from follow-up visits of children who failed the initial screening.

**Findings:**

This study included 452 children aged 7–13 years old. Based on audiograms, screening showed that 123 (27.2%) of the children had hearing impairment. The study has important implications for clinical practice and health policy. There is a need for systematic monitoring of hearing status among children of this age, and parents and educators need to be made aware of the significance of hearing loss.

## Introduction

Around 466 million people worldwide have disabling hearing disorders, and 34 million of these are children (World Health Organization, 2019). It is well known that late detection, and hence delayed therapy and rehabilitation of hearing disorders, has negative consequences in terms of language and speech development, emotional and cognitive development, and learning at all levels (Skarżyński and Ludwikowski, 2018). For these reasons, universal newborn hearing screening programs have been introduced in many countries to allow early identification of hearing loss (Fortnum *et al.*, 2001; Skarżyński *et al.*, 2014).

To devise suitable intervention strategies for a patient, an important aspect is obtaining precise objective auditory data (Ciorba *et al.*, 2013). In many countries, however, there is a lack of diagnostic follow-up, making continuous care difficult (Govender *et al.*, 2015). Although there is increasing awareness of hearing loss and its sequelae, prevention and treatment are still not regarded as urgent, especially in the lowest income countries. In India, there is no routine hearing screening test for children (Vaidyanath and Yathiraj, 2014; Ramkumar, 2017). Kanji *et al.* (2018) showed that in South Africa, there are still many barriers to efficient infant hearing screening. Shinn *et al.* (2019) reported that in rural areas of Kenya, ambient noise levels during hearing screening were so high that there were many false-positive referrals. In developing countries, the high cost of equipment, poor availability of hearing services, long distances, and shortage of professionals inevitably lead to shortfalls in hearing health care (Sandström *et al.*, 2020).

There is a significant difference in the prevalence of ear diseases between developed and developing countries (Jacob *et al.*, 1997). In both, the most frequent causes of hearing loss are conductive and treatable. According to WHO (2019), 60% of hearing loss in children is due to preventable causes. Epidemiological data from regions with low gross national income show that the prevalence of hearing impairment in children and adults is twice that as in high-income countries (Harris and Dodson, 2017). Poverty and unemployment make matters worse. We conclude that hearing screening and early intervention should be widely promoted in developing countries; such an effort should be rewarded with better educational outcomes.

In developing countries like Kyrgyzstan, hearing screening programs do not exist. Implementing them is extremely challenging due to long-standing health disparity issues. A major one is a basic lack of funding for health programs (Thomas *et al.*, 2014). This is aggravated by a shortage of audiologists, lack of awareness of the benefits of hearing screening, and the unavailability of equipment such as audiometers, auditory brainstem response machines, and otoacoustic emission equipment. In this situation, a low-cost hearing screening test could be a positive first step in improving the hearing health of people from impoverished areas. Such an approach requires low-cost procedures that can be supplied to areas where technological or human resources are financially out of reach. If groups at risk of hearing disorders can be identified at the earliest possible stage, then that would help reduce health inequalities (Botasso *et al.*, 2015).

A way to solve the problem is to use teleaudiology technology to perform routine screening tests at low cost. Teleaudiology has the potential to improve health services in developing countries by connecting hearing specialists, such as audiologists or laryngologists, with hearing-impaired patients in remote locations. Without teleaudiology, these patients would face impossible geographical and economic barriers (Bush *et al.*, 2016). Services for remote areas can include hearing screening and also later follow-up care.

Generally, two methods can be used for teleaudiology: on-site and remote. There is evidence that remote hearing screening gives comparable results with on-site screening. The two methods were compared in American children. Testing was done on the same children twice: once by on-site screening and again by telemedicine. For pure-tone audiometry, no statistically significant differences were found (Lancaster *et al.*, 2008). Choi *et al.* (2007) compared on-site audiometry using a personal computer (PC)-based audiometer with remote testing over the Internet on 12 adult subjects with normal hearing. Comparison between face-to-face audiometry with a PC-based system and on a conventional audiometer showed that 96.3% of the results did not differ by more than 5 dB. Givens and Elangovan (2003) compared pure-tone air thresholds (0.25–8 kHz) determined through remote synchronous audiometry on 45 subjects. Statistically, there was no significant difference between the remote test results and the face-to-face tests. The results of the study are in line with the studies by Lancaster *et al.* (2008) and Ciccia *et al.* (2011) who reported no differences between in-person and tele-pure tone audiometry in school children. A study conducted by Śliwa *et al.* (2011) indicated that systematic hearing screening of school-age children is essential and as important as those for newborns. In this way, children with hearing disorders can be identified in a timely manner.

In this study, audiometric hearing screening was performed using the Platform for Sensory Organs Examination. This tool is based on the asynchronous telemedicine model which allows screening tests to be performed and comprehensively analyzed. The system uses the System of Integrated Communication Operations (SZOK), which has been implemented in many European, African, and Asian countries (Skarżyński *et al.*, 2014: 2016). This screening model allows tests to be conducted by a trained assistant, with the results later evaluated by an audiologist or otolaryngologist at a central location. The present study was designed to detect hearing disorders in children in Kyrgyzstan using these teleaudiology tools. Any pupils with positive results were referred for diagnostic tests.

## Methods

### Participants

The study was conducted in two public primary schools in Bishkek, Kyrgyzstan. The schools were nominated by local authorities and approval from school management was obtained. Prior to testing, the children’s parents were informed of the testing procedures and signed a consent form for their children to participate in a hearing screening examination. If the parents gave written consent, their child was invited to be examined; all children willingly participated. Testing involved 452 children: 289 (63.9%) aged 7–8 years old and 163 (36.1%) aged 11–13 years.

### Audiometric measurement

Pure-tone audiometric testing was conducted using the Platform for Sensory Organs Examination. Pure-tone audiometry is the gold standard for hearing screening programs for school-age children (Honeth *et al.*, 2010; Masalski *et al.*, 2018). The platform was developed by the Institute of Sensory Organs in collaboration with the Institute Physiology and Pathology of Hearing. The system is based on a powerful central computer and many portable computers communicating with it via the Internet. Each portable device is equipped with software that allows it to perform pure-tone audiometry. The platform carries Sennheiser HDA200 headphones which provide effective acoustic isolation of the ear from background noise.

The platform allows air conduction audiometry testing to be performed for each ear separately over a frequency range of 0.5–8 kHz. It is limited to hearing thresholds below 80 dB HL. The Hughson and Westlake procedure of threshold measurement is used (i.e., two out of three responses at threshold are required; Yantis, 2002). The platform has been found to be an effective and accurate tool for testing hearing (Śliwa *et al.*, 2011) and has been validated as part of a telemedicine model (Skarzynski *et al.*, 2016). The equipment was calibrated according to PN-EN ISO 389-1:^2002^. Testing was performed by three experienced audiologists.

The results of audiometric hearing tests were automatically collected in a central database ‘SZOK’®. The collected results were marked with a unique identifier, which is guaranteed to fully protect a subject’s personal data in accordance with applicable law. Audiometry testing was conducted during school hours in a quiet room and was stopped when pupils had a break. The test environment was controlled according to PN-EN ISO 8253-1/2005.

### Analysis criteria

A positive (i.e., refer) test result was taken to be an air conduction threshold value higher than 20 dB HL at one or more frequencies in at least one ear (Clark, 1981; Bess, 1985; Niskar *et al.*, 1998). Unilateral hearing loss was recognized when there was normal hearing in one ear and hearing loss in the other ear with a threshold higher than 20 dB HL at one or more frequencies. Thresholds in both ears higher than 20 dB HL at one or more frequencies were defined as bilateral hearing loss. Each audiogram with a positive result was divided into one of three types (Skarżyński *et al.*, 2014: 2016):

- *Low-frequency hearing loss* (LFHL), in which the hearing threshold for 500 and/or 1000 Hz was above 20 dB HL, while the threshold for other frequencies did not exceed 20 dB HL.
- *High-frequency hearing loss* (HFHL), in which the hearing threshold for 4000 and/or 8000 Hz was above 20 dB HL, while the hearing threshold for other frequencies did not exceed 20 dB HL.
- *Other*: abnormal screening results in which the hearing threshold was greater than 20 dB HL at two or more frequencies.

### Statistical analysis

A chi-square test for independence was conducted to determine if there was a significant association between age and the results of hearing screening. A *z*-test for the equality of two proportions was made to compare rates of various types of hearing loss. Statistical significance was specified as a *P*-value less than 0.05. Analysis was conducted using IBM SPSS Statistics v. 24.

## Results

Positive results of hearing screening were obtained in 123 children (27.2%), while the other 329 children (72.8%) had audiometric thresholds below the 20 dB criterion. There were 80 children (65% of 123 children with positive outcome) who had unilateral impairment and 43 children (35%) who had bilateral impairment. The data divided into age groups are presented in Table 1.

**Table 1.** Numbers (and percent) of positive results of hearing screening

	Number of children	Positive results	Unilateral	Bilateral
7–8 years	289	93 (32.2%)	62 (66.7%)	31 (33.3%)
11–13 years	163	30 (18.4%)	18 (60.0%)	12 (40.0%)
Total	452	123 (27.2%)	80 (65.0%)	43 (35.0%)

There was a statistically significant difference in the frequency of positive results between the younger and older children: χ^2^ = 9.98; *P* = 0.002. Positive results were found more often in the younger children (32.2%) than in the older children (18.4%).

There was no statistically significant difference in the laterality of positive results between the younger and older children: χ^2^ = 0.44; *P* = 0.505. Positive results in one ear were more frequent than in both ears, regardless of age.

Considering ears which had hearing thresholds above the 25 dB criterion, there were 166 in total, including 78 ears with HFHL (47%), 12 ears with LFHL (7.2%), and 76 ears with other type of hearing loss (HL). The data divided into age groups are presented in Table 2.

**Table 2.** Frequency of different types of audiograms among 166 ears with a positive hearing screening result

	Ears with positive result	LFHL	HFHL	Other
7–8 years	124	6 (4.8%)	64 (51.6%)	54 (43.6%)
11–13 years	42	6 (14.3%)	14 (33.3%)	22 (52.4%)
Total	166	12 (7.2%)	78 (47.0%)	76 (45.8%)

LFHL = low-frequency hearing loss; HFHL = high-frequency hearing loss

HFHL was more frequent than LFHL both in the younger group (*P* < 0.001) and in the older group (*P* = 0.041). The rate of HFHL was significantly higher in younger children than in older children (*P* = 0.040). The rate of LFHL was significantly higher in the older children than in the younger group (*P* = 0.039). The ratios of other types of HL were similar in both age groups (*P* = 0.323).

### Follow-up

Parents of children with positive results of hearing screening were provided with information that the child should be referred for specialist diagnostics. Information on how many of the 123 children with positive results received follow-up testing and intervention services was not available, but we did manage to collect follow-up data from 27 children with positive results. These children came to a pediatrician or otolaryngologist in medical clinics cooperating with the hearing screening organizers.

In 21 of the 27 children with positive outcome of hearing screening, some hearing problem was found (i.e., the true positive rate was 78%). In nine cases, the ear canal was blocked with ear wax. After removing it, hearing was found normal. In five cases, otitis media was diagnosed and appropriate antibiotic treatment was implemented. Tympanoplasty was ordered in two children due to perforation of eardrum. In two children, hyperplasia of the pharyngeal tonsil was found and adenotomy was recommended. Two children had a recent infection of the respiratory system which could have affected hearing and the doctor decided to wait for a complete recovery. One child had otosclerosis which had been diagnosed earlier and was not declared by the parents before hearing screening. Hearing impairment was not confirmed in six children with positive outcome of hearing screening (i.e., there was a false-positive rate of 22% in the group of 27 children).

Three selected audiograms of children with follow-up data are presented below. The first child was diagnosed with an eardrum perforation in the left ear. The ENT specialist ordered a laboratory test to identify any bacterial infection, antibiotic drops were prescribed, and tonal audiometry was ordered. A second visit took place one month later. There was no bacterial infection in the left ear, but the eardrum was still perforated. Tympanoplasty in the left ear was ordered (Figure 1A). The second audiogram shows the audiogram of a child with earwax in both ears. There was decreased hearing in the right ear, probably because of more severe blockage in this ear. Excess wax was removed by a doctor, an audiological examination was performed, and hearing was found to be normal (Figure 1B). The third audiogram comes from a child diagnosed with chronic suppurative otitis media. It was treated with antibiotic drops. Otoscopy and tonal audiometry were performed after treatment, and hearing was found to be normal. The ENT specialist recommended monitoring and regular hearing tests (Figure 1C).

**Figure 1A.**
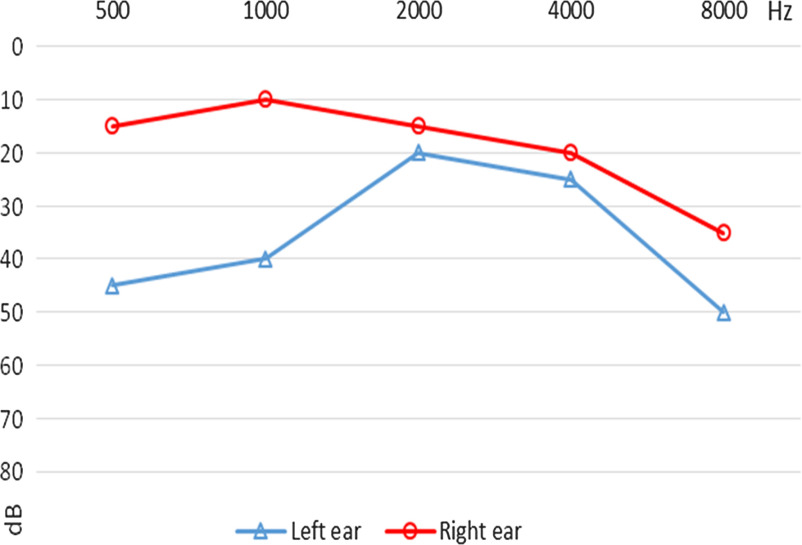
Audiogram of the child with eardrum perforation and tympanoplasty ordered (case #1).

**Figure 1B.**
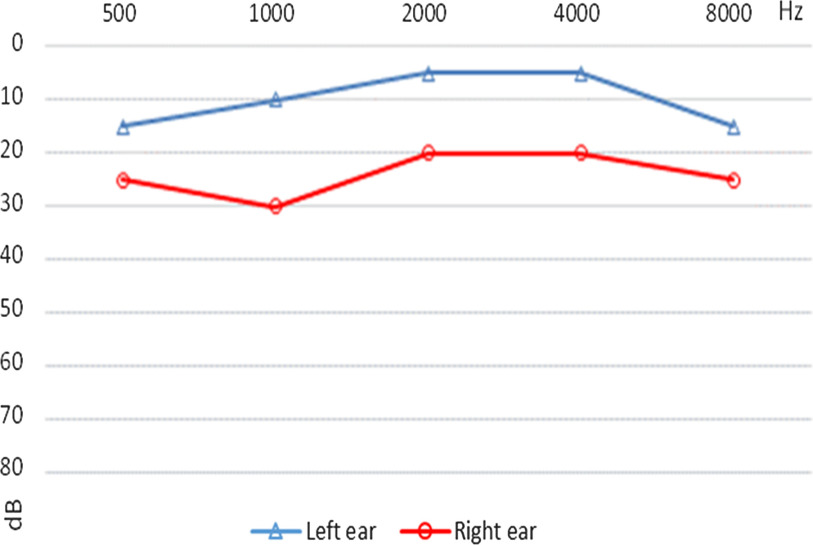
Audiogram of the child with earwax (case #2).

**Figure 1C.**
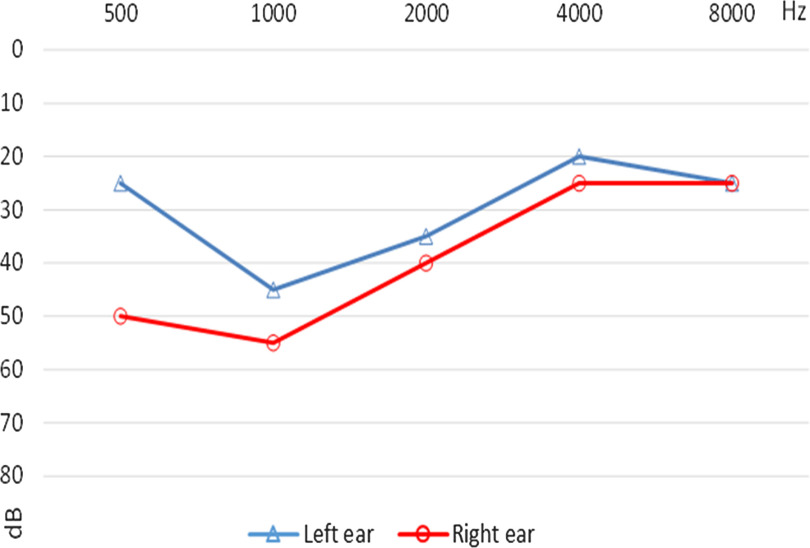
Audiogram of the child with acute otitis media (case #3).

## Discussion

The primary purpose of this study was to investigate the rate of hearing disorders in school-age children in Bishkek and, if tested positive, refer them for detailed diagnosis. An additional goal of the screening program was to alert parents and the school more generally to children’s hearing problems. These activities were part of a general effort to improve the state of medical care in Kyrgyzstan using information technology. The effectiveness of using an asynchronous model of screening for hearing impairment in children from primary school was also evaluated. The telemedical model of screening was aimed at improving the hearing health services for children in Kyrgyzstan where long-distance travel to regional or city centers is often difficult and sometimes impossible.

In Kyrgyzstan, screening for newborn and school-age children is not done due to lack of equipment and qualified personnel. The limited access to specialist doctors is associated with a high percentage of people with various hearing disorders. In this study, the observed prevalence of hearing impairments was 27.2%. This figure is similar to other studies conducted in Tajikistan, where hearing impairment was found in 23.7% of the surveyed school-age children (Skarzyński *et al.*, 2016). In the study conducted by Niskar *et al.* (1998), almost 15% of children aged 6–11 years had positive screening results. Feder *et al.* (2017) found that 7.7% of Canadian children aged 6–19 had HL at one or more pure-tone frequencies. Govender and Mars (2018) assessed 146 ears and found that 23 ears of 20 children (16%) presented with hearing loss. In rural areas of Poland, the rate of positive results of hearing screening was 16.4% (Skarżyński *et al.*, 2020). The variability in prevalence may be explained by different sample numbers, different evaluation protocols (Tarczyński and Piotrowska, 2016), and by the various ages of the children. In addition, the prevalence of hearing loss in children in developed countries is typically lower than in developing countries (Mahomed-Asmail *et al.*, 2016). Fortnum *et al.* (2001) suggested that reasons for the differences include the absence of hearing screening programs, the impact of poverty and malnutrition, stigma, lack of education about hearing disorders, and limited access to health care in developing countries.

Our results indicate that 7.2% of children with positive screening results had a LFHL. Data from an American study indicate a similar incidence of LFHL – 7.1% (Bess, 1985). A higher incidence of this type of hearing disorder was reported in a Polish study (Skarżyński *et al.*, 2019), where 23% of the screened children were classified as having LFHL. In a Nigerian study (Oyewumi and Adejumo, 2011), 33.4% (167 out of 500 examined children) were found to have LFHL in their right ear and 7.8% in their left. Similar data have been reported in Tajikistan, where 34% of children were diagnosed with LFHL (Skarzyński *et al.*, 2016). In some cases, a LFHL may be temporary and, depending on the individual case, pharmacological or surgical intervention may be effective. One of the most common reasons for temporary LFHL is inflammation of the middle ear. Otitis media with effusion is one of the most common childhood diseases (Minovi and Dazert, 2014). The delayed detection of otitis media with effusion in young children is a serious matter. Another reason for temporary hearing loss is upper respiratory tract infection (URTI). Czech *et al.* (2011) observed that children suffering from URTI often have temporary conductive hearing loss. As with congenital malformations, the benefits of early intervention in children with otitis media with effusion far outweigh the cost of screening, which provides good justification for conducting them (Hunt *et al.*, 2017).

In this study, 47% of the children with positive results were diagnosed with HFHL. A similar incidence of HFHL – 43.9% – was obtained in a Polish study (Skarżyński *et al.*, 2019). In comparison, the hearing screening in Tajikistan found that the percentage of children with HFHL was 25.5% (Skarzyński *et al.*, 2016). Children with HFHL may appear normal, but they may experience difficulties in many situations. For example, they may seem distracted because of a difficulty in understanding speech in a noisy background. In HFHL, speech disorders and articulation problems can also arise. It is important that children with HFHL should be permanently supported in school and in their home environment (Stelmachowicz *et al.*, 2004).

Unilateral hearing loss (65%, 80/123) was more common than bilateral losses, in line with results reported by Skarzyński *et al.* (2019). A unilateral hearing loss can affect many areas of a child’s development, can cause difficulties in sound source location, and problems with perceiving speech in background noise. In addition, there can be problems associated with loss of binaural summation and sound localization, causing delays in speech-language development and impairments to school performance (Skarżyński and Ludwikowski, 2018). On this basis, identification of hearing loss, whether unilateral or bilateral, calls for effective management so as to minimize these adverse effects (Grandpierre *et al.*, 2018).

In the current study, we were only able to collect follow-up data from 27 children who had a positive result. The low follow-up rate among students referred from the school suggests a low level of support for the program from caregivers. This could be because of lack of knowledge about hearing disorders or problems with traveling to the medical center. Engagement of parents and school personnel with the school screening program is essential if the prevalence of treatable ear diseases and associated hearing disorders in children is to be reduced.

The data from the present study suggest that it is possible to use a telemedicine model to assess the hearing status of school-age children and to provide long-distance expert assistance. It is necessary to train local medical staff to perform hearing screening. However, health care personnel involved with hearing services in less modern locations need consistent training, oversight, and feedback by experienced audiologists in order to provide quality services. Hearing screening opens up the possibility of detecting hearing problems and then directing the children to further specialist diagnostic evaluation and intervention.

## Limitation

The present study was confined to the capital city of Kyrgyzstan, and it is difficult to generalize the findings to the whole pediatric population of the country. Moreover, only air conduction thresholds were measured; there was no bone conduction, otoscopy, tympanometry, or otoacoustic emission measurements.
